# Predictive Value of Red Cell Distribution Width-to-Platelet Ratio Combined with Procalcitonin in 28-day Mortality for Patients with Sepsis

**DOI:** 10.1155/2024/9964992

**Published:** 2024-08-12

**Authors:** Ying Si, Bo Sun, Yongmao Huang, Ke Xiao

**Affiliations:** ^1^ Department of Infectious Diseases Department of Tuberculosis, Infection, and Immunity Laboratory The Affiliated Hospital of Southwest Medical University, Luzhou, Sichuan, China; ^2^ Department of Ultrasound The Affiliated Hospital of Southwest Medical University, Luzhou, Sichuan, China

## Abstract

**Objectives:**

The objective of this study was to investigate the predictive value of erythrocyte distribution width-to-platelet ratio (RPR) combined with procalcitonin (PCT) on 28-day mortality in patients with sepsis.

**Methods:**

A total of 193 patients with sepsis admitted to the Affiliated Hospital of Southwest Medical University from January 2013 to January 2018 were selected as the study objects. Univariate and multivariate analyses were used to understand the indicators related to the 28-day prognosis of patients, and the ROC curve was further drawn. The Kaplan–Meier curve was used to evaluate the prognosis of patients.

**Results:**

A total of 193 patients were enrolled and divided into the survivor group (=156) and nonsurvivor group (=37) according to the prognosis within 28 days. The median age was 62.5 years, and 64.7% were males. Multivariate analysis showed that PCT and RPR were independent risk factors for 28-day prognosis in sepsis patients. The area under the ROC curve of PCT and RPR were 0.894 and 0.861, respectively, and the cutoff values were 27.04 and 0.12, respectively. Survival curve analysis showed that PCT and RPR were associated with the 28-day prognosis of patients, and the combination of PCT and RPR had a better predictive effect.

**Conclusions:**

PCT and RPR are independent predictors of sepsis prognosis. The combined application of PCT and RPR (PCT-RPR) can further improve the predictive performance and provide a reference for the clinical diagnosis, treatment, and prognosis evaluation of sepsis patients.

## 1. Introduction

Sepsis is a clinical syndrome with rapid disease progression and high mortality, and the treatment is difficult [1]. Sepsis results in a dysregulated organ response to infection, which further leads to organ dysfunction [2]. Therefore, screening of risk factors related to the prognosis of sepsis is of great significance for clinical diagnosis and treatment and improvement of prognosis.

The ratio of red blood cell distribution width to platelet (RPR) calculated based on the ratio of red blood cell distribution width to platelet is an effective prognostic factor reflecting the status of systemic inflammation [3]. Studies have reported that RPR is closely related to the prognosis of sepsis [4]. Ge et al. [5] found that RPR is an effective predictor of the prognosis of adult sepsis. Procalcitonin (PCT) is an important clinical indicator for the evaluation of sepsis patients and the guidance of drug use [6]. Studies have shown that PCT is closely related to 28-day mortality in patients with sepsis [7].

Both RPR and PCT are associated with the prognosis of patients with sepsis. However, there are no reports of RPR combined with PCT to predict 28-day mortality in sepsis patients. Therefore, the purpose of this study was to explore the predictive value of RPR and PCT in patients with sepsis and further understand the predictive effect of RPR combined with PCT in patients with sepsis.

## 2. Methods

### 2.1. Patients and Selection Criteria

A total of 193 patients with sepsis who were treated in the Affiliated Hospital of Southwest Medical University from January 2013 to January 2018 were selected as the study objects. The inclusion criteria were as follows: (1) adult patients who met the diagnostic criteria for sepsis at admission [8] and (2) complete clinical data. The exclusion criteria are as follows: (1) those under the age of 18, (2) accompanied by blood system disease, and (3) patients with sepsis during hospitalization. Peripheral blood samples were collected from patients diagnosed with sepsis, and serum PCT level, red blood cell distribution width, and serum platelet level were detected. RPR was calculated by dividing red blood cell distribution width by platelet count. The patients were divided into survival and nonsurvival groups based on whether they died within 28 days of sepsis diagnosis. This study was reviewed and approved by the Ethics Committee of the Affiliated Hospital of Southwest Medical University (Grant no. KY2023299).

### 2.2. Data Collection

Data on sepsis patients were extracted from the hospital's electronic medical record system, a retrospective collection including age, sex, number of patients using vasoactive drugs, hospital-free days, ICU-free days, duration of mechanical ventilation, Sequential Organ Failure Assessment (SOFA) score, and underlying disease (coronary artery disease (CAD), kidney disease, hypertension, and diabetes). Collection of the first biochemical indicators taken at the time of the patient's admission to the hospital, biochemical markers, included lymphocyte, white blood cell (WBC), red blood cell distribution width (RDW), platelet (PLT), prothrombin time (PT), alanine aminotransferase (ALT), albumin, aspartate aminotransferase (AST), total bilirubin (TBil), procalcitonin (PCT), serum creatinine (SCr), and RPR. The main infection site of most sepsis patients is lung infection, followed by urinary system, skin soft tissue, hepatobiliary system, and abdominal cavity infection.

### 2.3. Statistical Analysis

SPSS 25.0 (IBM, Armonk, NY, USA) was used for data analysis. The ROC curve is used to determine the truncation values of the RPR and PCT. Categorical variables are expressed in counts and percentages, while median and quartile are used to describe qualitative variables. Univariate and multivariate analyses were used to screen the risk factors associated with the 28-day prognosis of patients with sepsis. The prognosis of different groups was analyzed by the Kaplan–Meier survival curve. *P* < 0.05 was considered statistically significant.

## 3. Results

### 3.1. Patient Demographics

**Figure 1:**
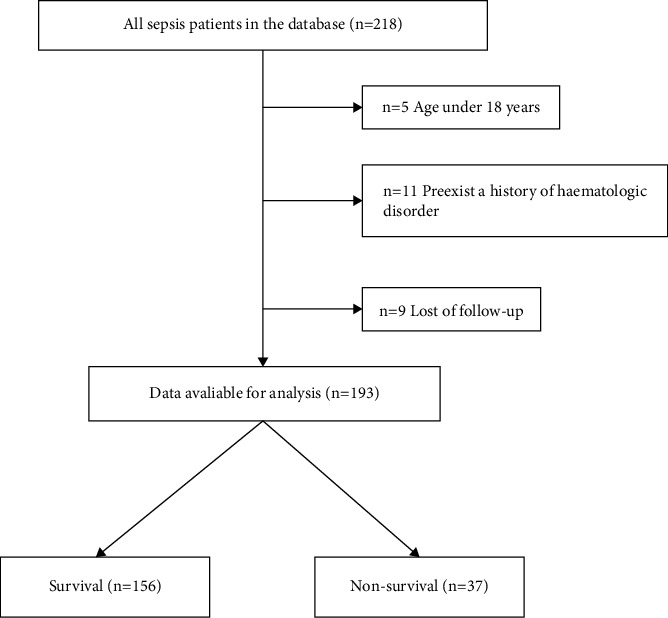
Flowchart for patient enrollment and study design.

A total of 193 patients were included in the study. Patients were divided into a survival group (*n* = 156) and a nonsurvival group (*n* = 37) based on their survival within 28 days after diagnosis of sepsis (Figure 1). The comparison of relevant clinical features between the two groups is shown in Table 1. Of these, 64.7% were men with a median age of 62.5 years. There were statistically significant differences in ICU-free days, SOFA score, CAD, lymphocyte count, platelet count, RDW, PT, albumin, TBil, SCr, PCT, and RPR between the two groups. Notably, hospital-free days stay in the nonsurviving group was significantly shorter than that in the surviving group. PCT and RPR values were generally higher in the nonsurviving group than in the surviving group.

### 3.2. Factors Associated with 28-Day Mortality in Multivariate Analysis

In order to further understand the independent risk factors affecting the prognosis of sepsis patients, a multivariate analysis of the abovementioned factors was conducted, and the results showed that SOFA score, CAD, RDW, PCT and RPR were independent risk factors for the prognosis of sepsis patients (*P* < 0.05) (Table 2).

### 3.3. ROC Curve Analysis of PCT, RPR, and PCT-RPR for 28-Day Mortality of Patients with Sepsis

**Figure 2:**
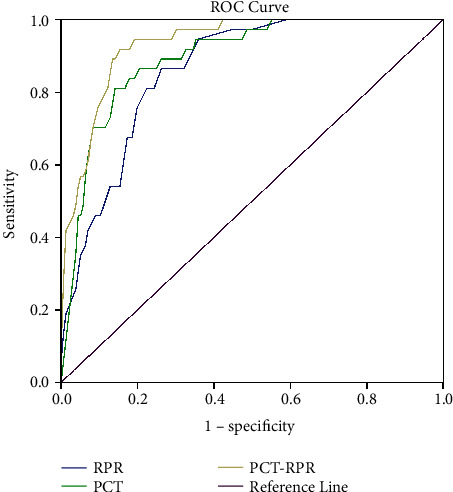
ROC curve analysis of PCT, RPR, and PCT-RPR for 28-day mortality of patients with sepsis.

The ROC curve was drawn according to the PCT and RPR values of sepsis patients at admission. The size of the area under the curve represented the predictive performance. The larger the area, the better the predictive performance. Our research results show that the AUC of the combination of PCT and RPR is 0.934, while the AUC of PCT and RPR alone is 0.894 and 0.861, respectively, indicating that the predictive performance of the combination of PCT and RPR is significantly better than that of a single indicator (Figure 2).

### 3.4. 28-Day Mortality Rate according to PCT or RPR

**Figure 3:**
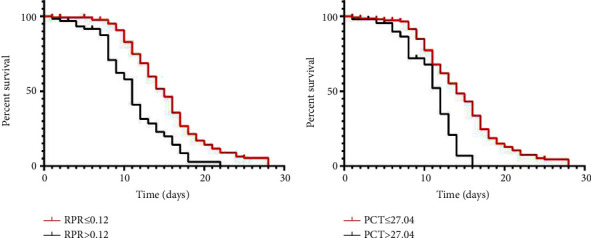
Kaplan–Meier survival curve of 28-day mortality according to the optimal cutoff of PCT = 27.04 and RPR = 0.12.

To further understand the predictive power of PCT and RPR for 28-day mortality in patients with sepsis, PCT and RPR were divided into two groups based on their cutoff values. Kaplan–Meier survival curve analysis showed that the 28-day mortality in PCT ≤ 27.04 and RPR ≤0.12 groups was significantly lower than that in PCT > 27.04 and RPR > 0.12 groups (Figure 3).

### 3.5. Predictive Value of PCT-RPR Combination for 28-Day Mortality in Patients with Sepsis

**Figure 4:**
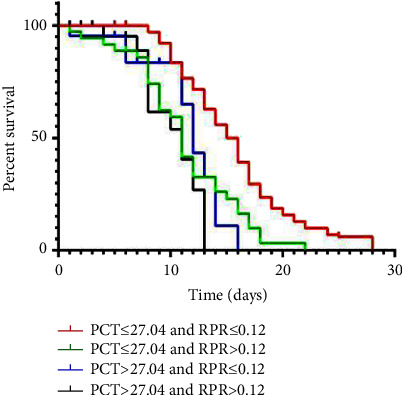
The combination of PCT and RPR was found to enhance prognostic accuracy for patients with sepsis.

All patients were divided into four groups according to different PCT and RPR values: (1) low PCT and low RPR groups; (2) low PCT and high RPR groups; (3) high PCT and low RPR groups; and (4) high PCT and high RPR groups. The results showed that patients in group 1 had the best prognosis, while patients in group 4 had the worst prognosis (Figure 4).

## 4. Discussion

Our study found that PCT and RPR were independent risk factors for 28-day prognosis in patients with sepsis. ROC curve analysis showed that the best cutoff values of PCT and RPR were 27.04 and 0.12, respectively. Notably, patients with higher PCT and RPR had a poorer 28-day prognosis. In addition, further analysis found that the combined prediction performance of PCT and RPR was better than that of a single indicator, and the survival curve showed that the low PCT and RPR groups had the best prognosis at 28 days.

Sepsis is an infectious disease with a high burden and high mortality, which easily leads to multiple organ dysfunction, with a mortality rate as high as 35.5% [9]. Although there have been some advances in the diagnosis and treatment of sepsis in recent years, the case fatality rate remains high. Studies have shown that red blood cells are more prone to abnormalities in inflammatory responses [10, 11]. RDW reflects the size of red blood cells in the body and can be used to diagnose anemia. However, a state of sepsis can also lead to elevated RDW in the body. In the state of sepsis, the hematopoietic function of patients is inhibited, resulting in impaired RBC maturation and elevated RDW [12]. Platelet count can effectively predict the prognosis of sepsis patients and identify patients with the worst prognosis [13]. The severe inflammatory response can lead to excessive consumption of coagulation factors and platelets, induce DIC, and ultimately lead to poor prognosis [14]. In summary, RPR calculated by the ratio of red blood cell distribution width-to-platelet number can reflect the status of red blood cells and platelets *in vivo* and can further predict the prognosis of patients with sepsis.

RPR is a simple and effective prognostic indicator of inflammation and is often used to predict the prognosis of infectious diseases [15–17]. In this study, the optimal critical value of RPR is 0.12, which is consistent with the previous critical value of RPR ranging from 0.06 to 0.14 [18, 19]. In our study, the multifactor analysis showed that RPR was an independent prognostic factor for sepsis patients, and further survival analysis showed that patients with low RPR had a poor prognosis, which was consistent with previous reports [20, 21].

PCT is a common and highly specific prognostic indicator for sepsis. Compared with traditional inflammatory indicators such as white blood cells and C-reactive protein, PCT has higher sensitivity and accuracy [22–24]. Previous studies have found that PCT is a stable and effective predictor of sepsis patients and can identify patients with poor prognosis [25–28]. In our study, PCT was not only an independent predictor of sepsis but was strongly associated with 28-day mortality in patients with sepsis.

As mentioned above, both RPR and PCT are closely related to the prognosis of patients with sepsis. PCT and RPR are the most commonly used and easily obtained prognostic evaluation indicators for sepsis patients and are widely used in clinical practice. So, we speculate that the combination of RPR and PCT may be more advantageous in predicting the prognosis of patients with sepsis than a single indicator.

In our study, ROC outcome statistics showed that combined diagnosis achieved the largest AUC than single biomarker diagnosis, indicating that combined diagnosis has a higher accuracy. Further survival analysis showed that the PCT ≤ 27.04 and RPR ≤ 0.12 groups had the best prognosis. Therefore, we speculate that the combination of RPR and PCT may be a potentially useful indicator of the prognosis of patients with sepsis and can be used as a routine screening tool to develop personalized protocols for different patients. Patients admitted to the hospital with sepsis were risk stratified by PCT and RPR thresholds found in this study. For patients with RPR > 0.12 and PCT > 27.04, by strengthening the observation and nursing of the disease changes, the incidence of severe complications such as septic shock and multiple organ dysfunction can be reduced as much as possible, so as to improve the prognosis of patients.

However, this study has several limitations. First, our overall sample size is relatively small. Second, we only studied the relationship between the relevant indicators and the short-term prognosis of patients. Whether the relevant indicators are related to the long-term prognosis of patients need further research. Finally, RPR, PCT, and SOFA scores are independent prognostic factors for sepsis patients, and the relationship between the three factors and the prognosis of sepsis can be further explored in the later stage, which is conducive to a more comprehensive understanding of the prognosis of sepsis. However, we believe that the combination of RPR and PCT may be an effective predictor of prognosis in patients with sepsis with further research in multi-institution and multisample.

## 5. Conclusions

In conclusion, our study identified RPR and PCT as independent predictors of prognosis in patients with sepsis. As a novel, economical and reliable biomarker, PCT-RPR has potential application value in the diagnosis and prognosis of sepsis patients.

## Figures and Tables

**Table 1:** Characteristics of the patients with sepsis.

Variables	Survival (*n* = 156)	Nonsurvival (*n* = 37)	*P* value
Age (years)	62.0 ± 16.5	64.3 ± 17.9	0.469
Male, *n* (%)	100 (64.1)	25 (67.5)	0.692
Hospital-free days	10.3 (0, 55)	5.6 (0, 20)	0.056
ICU-free days	9.6 (0, 18)	5.1 (0, 24)	0.039
Number of patients using vasoactive drugs	112	22	0.782
SOFA score	3.76 ± 3.44	8.85 ± 4.22	0.021
Chronic comorbidities			
Hypertension (%)	35 (22.4)	14 (37.8)	0.053
Diabetes (%)	28 (17.9)	8 (21.6)	0.606
CAD (%)	11 (7.1)	8 (21.6)	0.013
Renal disease (%)	5 (3.2)	3 (8.1)	0.181
WBC count, mean ± SD (10^9^/L)	13.09 ± 8.33	15.26 ± 9.22	0.164
Lymphocyte count, mean ± SD(10^9^/L)	0.97 ± 0.75	0.67 ± 0.46	0.023
PLT count, median (IQR) (10^9^/L)	187.50 (33.00, 878.00)	102.00 (10.00,27.600)	<0.001
RDW, mean ± SD, (%)	14.43 (10.10, 19.73)	16.87 (13.93, 22.27)	<0.001
PT, mean ± SD, (S)	14.51 ± 3.99	16.62 ± 10.50	0.047
Albumin, median (IQR), (g/L)	34.20 (19.50, 48.10)	29.70 (20.00, 43.20)	0.005
ALT, median (IQR), U/L	29.45 (2.70, 337.80)	36.60 (4.40, 272.80)	0.239
AST, median (IQR), U/L	36.40 (7.80, 568.50)	57.90 (10.30, 292.60)	0.055
TBil median (IQR), *μ*mol/L	13.10 (3.30, 67.80)	15.20 (5.10, 67.50)	0.046
SCr, mean ± SD, *μ*mol/L	20.79 ± 14.82	183.55 ± 170.84	<0.001
PCT, mean ± SD, (ng/ml)	12.04 ± 22.24	60.41 ± 34.78	<0.001
RPR, mean ± SD	0.10 ± 0.075	0.31 ± 0.36	<0.001

**Table 2:** Factors associated with in-hospital mortality in multivariate analysis.

Variables	B	Se	Wald	*P* value	HR	95% CI for HR	
						Lower	Upper
CAD	3.578	1.101	10.560	0.001	0.028	0.003	0.242
SOFA score	4.561	1.568	8.569	0.003	0.022	0.002	0.325
Lymphocyte count	0.545	0.532	1.047	0.306	0.580	0.204	1.647
PLT count	0.015	0.008	3.646	0.056	1.015	1.000	1.032
RDW	3.161	0.813	15.124	<0.001	0.042	0.009	0.208
PT	0.020	0.038	0.285	0.593	0.980	0.909	1.056
Albumin	0.040	0.051	0.605	0.437	1.040	0.942	1.149
TBil	0.021	0.023	0.801	0.371	0.980	0.936	1.025
SCr	0.004	0.003	2.473	0.116	0.996	0.990	1.001
PCT	3.412	0.744	21.044	<0.001	0.033	0.008	0.142
RPR	2.264	0.678	11.158	0.001	0.104	0.028	0.392

## Data Availability

The original contributions presented in the study can be inquiried and directed to the corresponding authors.
